# Unraveling molecular interconnections and identifying potential therapeutic targets of significance in obesity-cancer link

**DOI:** 10.1016/j.jncc.2024.11.001

**Published:** 2024-11-28

**Authors:** Alanoud Abdulla, Hana Q. Sadida, Jayakumar Jerobin, Imadeldin Elfaki, Rashid Mir, Sameer Mirza, Mayank Singh, Muzafar A. Macha, Shahab Uddin, Khalid Fakhro, Ajaz A. Bhat, Ammira S. Al-Shabeeb Akil

**Affiliations:** 1 Department of Human Genetics-Precision Medicine in Diabetes, Obesity and Cancer Research Program, Sidra Medicine, Doha, Qatar; 2 Qatar Metabolic Institute, Academic Health System, Hamad Medical Corporation, Doha, Qatar; 3 Department of Biochemistry, Faculty of Science, University of Tabuk, Tabuk, Saudi Arabia; 4 Department of Medical Laboratory Technology, Prince Fahad Bin Sultan Chair for Biomedical Research, Faculty of Applied Medical Sciences, University of Tabuk, Tabuk, Saudi Arabia; 5 Department of Chemistry, College of Sciences, United Arab Emirates University, Al-Ain, United Arab Emirates; 6 Department of Medical Oncology (Lab.), Dr. BRAIRCH, All India Institute of Medical Sciences (AIIMS), New Delhi, India; 7 Watson-Crick Centre for Molecular Medicine, Islamic University of Science and Technology, Pulwama, Jammu and Kashmir, India; 8 Translational Research Institute, Academic Health System, Hamad Medical Corporation, Doha, Qatar; 9 Laboratory of Animal Research Center, Qatar University, Doha, Qatar; 10 Department of Human Genetics, Sidra Medicine, Doha, Qatar; 11 College of Health and Life Sciences, Hamad Bin Khalifa University, Doha, Qatar; 12 Department of Genetic Medicine, Weill Cornell Medicine, Doha, Qatar

**Keywords:** Obesity, Cancer risk, Tumor microenvironment, Inflammation, Therapeutic interventions, Gut microbiome

## Abstract

Obesity, a global health concern, is associated with severe health issues like type 2 diabetes, heart disease, and respiratory complications. It also increases the risk of various cancers, including melanoma, endometrial, prostate, pancreatic, esophageal adenocarcinoma, colorectal carcinoma, renal adenocarcinoma, and pre-and post-menopausal breast cancer. Obesity-induced cellular changes, such as impaired CD8^+^ T cell function, dyslipidemia, hypercholesterolemia, insulin resistance, mild hyperglycemia, and fluctuating levels of leptin, resistin, adiponectin, and IL-6, contribute to cancer development by promoting inflammation and creating a tumor-promoting microenvironment rich in adipocytes. Adipocytes release leptin, a pro-inflammatory substance that stimulates cancer cell proliferation, inflammation, and invasion, altering the tumor cell metabolic pathway. Adiponectin, an insulin-sensitizing adipokine, is typically downregulated in obese individuals. It has antiproliferative, proapoptotic, and antiangiogenic properties, making it a potential cancer treatment. This narrative review offers a comprehensive examination of the molecular interconnections between obesity and cancer, drawing on an extensive, though non-systematic, survey of the recent literature. This approach allows us to integrate and synthesize findings from various studies, offering a cohesive perspective on emerging themes and potential therapeutic targets. The review explores the metabolic disturbances, cellular alterations, inflammatory responses, and shifts in the tumor microenvironment that contribute to the obesity-cancer link. Finally, it discusses potential therapeutic strategies aimed at disrupting these connections, offering valuable insights into future research directions and the development of targeted interventions.

## Graphical abstract

**Figure:**
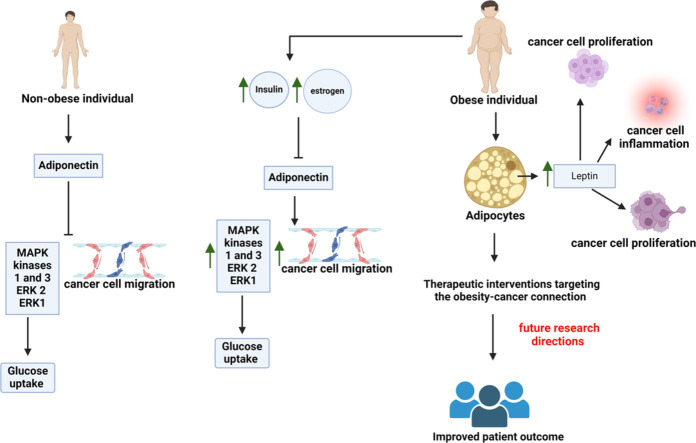

## 1 Introduction

Obesity is a growing global epidemic that affects millions of people worldwide and is characterized by an excessive accumulation of body fat. This buildup often results from an interplay between high caloric intake, sedentary lifestyles, genetic predispositions, and other psychosocial factors that modulate the balance of energy consumption and expenditure. It affects individuals across age groups, socio-economic backgrounds, and geographical regions worldwide. An illustrative study from the United States (US) highlighted the significant difference in weight trends among individuals based on their activity levels: those who consistently engaged in physical activities lost weight, whereas their less active counterparts experienced weight gain.^1^ According to the World Health Organization (WHO), global adult obesity rates have more than doubled since 1990, while rates of adolescent obesity have quadrupled.^2^^,^^3^ Clinically, the body mass index (BMI) is a pivotal gauge of obesity. Those with a BMI ranging from 30 kg/m^2^ to 35 kg/m^2^ are categorized as obese, whereas a BMI of 35 kg/m^2^ or more demarcates severe obesity.^4^ This metric isn't just a numerical value; it bears a weighty prognostic significance. Obesity is intricately linked to various metabolic dysregulations, such as insulin resistance, chronic inflammation, and altered adipokine secretion.^5^ Obesity also stands as a formidable precursor to a suite of medical complications, from type 2 diabetes mellitus, metabolic syndrome, and cardiovascular diseases to respiratory disorders.^6^^,^^7^

Further, disease susceptibility appears to scale with BMI, especially as individuals with a BMI exceeding 29, coupled with a substantial waist circumference, confront heightened risks of hypertension and aberrant blood lipid profiles.^6^ While BMI is widely utilized on a population level due to its simplicity and accessibility, it indeed has significant limitations when applied to individual assessments, especially in studies that aim to establish a clear connection between obesity and cancer. BMI is an indirect measure that does not differentiate between fat mass and lean mass. As a result, individuals with the same BMI may have vastly different levels of adiposity, leading to potential misclassification in studies associating obesity with cancer risk.^8^^,^^9^ The normal ranges for BMI can vary significantly across different racial and ethnic groups due to genetic, environmental, and cultural factors.^10^ This variability underscores the importance of considering population-specific cutoffs when using BMI as an alternative for obesity in cancer research. BMI does not account for metabolic health, which is increasingly recognized as a critical factor in the obesity-cancer link.^11^ Individuals with the same BMI can have differing levels of metabolic dysfunction, which may more directly contribute to cancer risk. Based on these limitations, we emphasize the importance of integrating other measures of adiposity and metabolic health, such as waist-to-hip ratio, body fat percentage, and biomarkers of metabolic syndrome, in future research.^9^

Disturbingly, the shadow of obesity extends into the realm of oncology. It potentiates a significant risk for a gamut of malignancies, including but not limited to melanomas, breast (both general and post-menopausal), prostate, pancreatic, colorectal, and renal carcinomas. It has been found to be a risk factor for cancer development and progression.^12^ The molecular interconnections between obesity and cancer, encompassing a complex network of signaling pathways, metabolic disturbances, cellular alterations, and inflammatory processes, have attracted more attention in recent years due to increasing epidemiological, clinical, and experimental evidence. Epidemiological studies have consistently demonstrated a positive correlation between obesity and the incidence of various cancer types, as well as poorer prognosis, increased cancer recurrence risk, and reduced overall survival rates.^13^ Notably, even as overall cancer incidences showcased a decline from 2010 to 2019 in the US, cancers tethered to obesity witnessed an upward trajectory, highlighting the detrimental impact of excess adiposity on cancer incidence and outcomes.^14^

At the cellular, metabolic, and systemic levels, obesity, characterized by excess adiposity, orchestrates a medley of disturbances. These encompass shifts in fatty acid dynamics, perturbations in the fat mass and obesity-associated protein (FTO) axis, and rerouting of glutamine and acetate pathways.^15^ It also intricately creates an environment conducive to tumorigenesis, cancer progression, and therapy resistance by fostering inflammation from excess adiposity.^16^ Beyond its role as an energy storage depot, adipose tissue is a dynamic endocrine organ capable of secreting various bioactive molecules known as adipokines.^17^ In individuals with obesity, there is a dysregulation in adipokine secretion, leading to chronic low-grade inflammation characterized by immune cell infiltration, insulin resistance, and aberrant signaling pathways implicated in cancer initiation and progression.^16^ Concurrently, obesity-induced changes in lipid metabolism lead to increased circulating levels of free fatty acids and dysregulation of cholesterol homeostasis, both of which have been linked with cancer cell proliferation, survival, and metastasis promotion.^18^ Hormones derived from adipose tissue, such as leptin and adiponectin, also exert pleiotropic effects on cancer cells, modulating cellular processes such as proliferation, apoptosis, angiogenesis, and immune surveillance.^19^^,^^20^ Hyperadiposity also leads to hypoxia, nutrient deprivation, and metabolic stress within the TME, promoting cancer cell survival and adaptation.^21^ Moreover, other obesity-associated alterations include extracellular matrix remodeling, immune cell infiltration, and angiogenesis, further fuel tumor growth and metastasis.^22^ Thus, the intricate interplay between obesity and cancer is multifaceted and involves a complex network of molecular and physiological changes.

Chronic inflammation is a hallmark feature of obesity and a critical mediator of obesity-associated cancer risk.^23^ Adipose tissue inflammation is characterized by the infiltration of immune cells, including macrophages, T cells, and neutrophils, which secrete pro-inflammatory cytokines and chemokines that foster tumorigenesis and progression.^19^ The activation of pro-inflammatory signaling pathways, such as nuclear factor-kappa B (NF-κB) and signal transducer and activator of transcription 3 (STAT3), in the tumor microenvironment promotes cancer cell proliferation, survival, angiogenesis, and metastasis.^19^ Obesity fundamentally disrupts cellular homeostasis, leading to several metabolic and immunological anomalies. Key among these are impaired CD8^+^ T cell function, dyslipidemia, elevated cholesterol levels, mild hyperglycemia, and insulin resistance. These disruptions are accompanied by altered circulating levels of critical markers such as leptin, resistin, adiponectin, and IL-6.^19^^,^^20^ Collectively, these factors create a pro-inflammatory and immunosuppressive environment that exacerbates disease progression.

The profound impact of these metabolic changes was vividly demonstrated in a study that compared the effects of high-fat diets with controlled diets in mice. The high-fat diet not only induced obesity but also significantly impaired the function of CD8^+^ T cells within the tumor microenvironment. This impairment weakened the immune system's ability to target and eliminate tumor cells, thereby facilitating tumor growth and progression.^24^ Tumor cells, in their effort to evade immune surveillance, engage in sophisticated metabolic reprogramming. They restrict blood flow to solid tumors, sequester essential nutrients, and remodel the surrounding metabolic landscape, creating a hostile environment for intratumoral T cells. This metabolic hijacking deprives T cells of the resources they need to function effectively, further compromising immune responses.^25^ Activated T cells, which are crucial for mounting an effective anti-tumor response, rely on specific metabolic pathways to sustain their proliferation and effector functions.^26^ In the context of obesity, the increased caloric intake and altered metabolic conditions may inadvertently provide tumors with an abundant supply of nutrients, fueling their growth and survival. However, the relationship between nutrition and tumor biology is complex and bidirectional. An intriguing study conducted in Italy explored the effects of a fasting-mimicking diet in breast cancer patients. This dietary regimen, when used in conjunction with conventional antitumor therapies, led to a significant reduction in blood glucose, insulin, and insulin-like growth factor 1 levels. Notably, this approach also triggered a substantial increase in the infiltration of CD8^+^ T cells and macrophages into the tumor, enhancing the immune system's ability to combat the disease.^27^

These findings underscore the intricate interplay between metabolism, immune function, and tumor progression, highlighting the potential of targeted nutritional interventions to modulate the tumor microenvironment and improve therapeutic outcomes.

This paper comprehensively reviews the molecular mechanisms underlying the obesity-cancer link, highlighting key signaling pathways, metabolic alterations, tumor microenvironment dynamics, and inflammatory processes implicated in cancer initiation, progression, and metastasis. We also highlighted the role of gut microbiome on the obesity-cancer link. Furthermore, we discuss emerging therapeutic targets that hold promise for disrupting obesity-driven oncogenic pathways and improving cancer outcomes in patients with obesity. By elucidating the complex molecular landscape of obesity-associated cancer, we aim to provide insights into personalized approaches to cancer management and pave the way for developing novel therapeutic interventions targeting the obesity-cancer axis.

We conducted a comprehensive literature search for this review across three major research databases: PubMed, ScienceDirect, and Web of Science. The search covered the period from database inception through April 20, 2024. Our goal was to identify reviews that explore the molecular interconnections between obesity and cancer. We utilized a predefined search algorithm with the following key terms: “Obesity,” “Cancer,” “Adipokines,” “Adipose Tissue,” “Molecular Mechanisms,” and “Interconnections,” including variations and related terms. The search was restricted to literature reviews published from 2014 onwards to ensure the inclusion of recent and relevant studies. Additionally, we manually screened the reference lists of the identified studies to locate further relevant literature that may have been missed in the initial database search. Since this is a narrative review, our methods were designed to identify and discuss a wide range of relevant studies, and they do not follow the rigorous protocols required for a systematic review. Specifically, we did not perform a systematic assessment of the quality or risk of bias of the included studies, nor did we apply predefined inclusion or exclusion criteria based on methodological quality. This approach ensured a thorough and up-to-date review of the current understanding of the molecular connections between obesity and cancer and potential therapeutic targets.

## 2 Obesity's association with cancer development

The intricate relationship between obesity and cancer has long been a topic of medical exploration. Researchers estimate that obesity plays a role in approximately 20 % of all cancers, highlighting its significance in global cancer epidemiology. There is an emerging consensus in the medical community that maintaining a stable weight can protect against certain cancers, including prostate and post-menopausal breast cancer.^28^^,^^29^ The varying degrees of association between obesity and different cancers indicate the complexity of metabolic and hormonal pathways involved. It's crucial to differentiate between correlations and causations in this context, especially when considering therapeutic and preventive strategies.

In this review, we define obesity-associated cancers as those for which there is substantial epidemiological evidence showing a positive correlation between obesity (as measured by BMI, waist circumference, or other markers of adiposity) and an increased risk of cancer incidence. Additionally, we consider tumors where obesity has been shown to negatively impact prognosis, including overall survival and cancer-specific survival. This dual criterion ensures that we capture both the increased risk and the worsened outcomes associated with obesity. For the specific cancers in our review, we relied on large-scale epidemiological studies and meta-analyses that consistently reported a positive association between obesity and these tumor types. For example, cancers of the breast (postmenopausal), colon, endometrium, kidney, liver, and pancreas are well-documented in the literature as having strong links with obesity both in terms of increased incidence and poorer prognosis.^12^^,^^30^ We also considered guidelines and reports from authoritative bodies such as the WHO and the International Agency for Research on Cancer (IARC), which classify these cancers as obesity-related.^31^

Based on the variability in findings across studies, while some research supports a strong link between obesity and certain cancers, other studies may show no relationship or even a negative correlation. The possible reasons for these discrepancies could be due to differences in study design, population heterogeneity, and the use of different obesity measures. We emphasize that our review presents a consensus based on the most robust and consistent evidence available, but we also recognize the limitations and ongoing debates in the field. There is a need to call for more standardized and rigorous research methodologies to better define and understand obesity-associated cancers. We suggest that future studies should attempt to integrate both incidence and prognosis data, consider a wider range of adiposity measures beyond BMI, and account for confounding factors such as metabolic health and lifestyle variables.

### 2.1 Breast cancer

Breast cancer, which is the most prevalent cancer among women worldwide and the second leading cancer-related death, has experienced a 0.5 % annual increase in incidence from 2010 to 2019. However, there has been a decrease in mortality rates from 2011 to 2020.^32^, ^33^, ^34^ The Million Women Study, which involved 1.2 million UK women aged between 50 and 64 years, found that 45,037 of these women were diagnosed with breast cancer. The study revealed that obesity increased the risk of developing postmenopausal breast cancer by approximately 30 %.^35^ This finding is supported by a meta-analysis of 34 studies, which established a correlation between higher BMI and postmenopausal breast cancers. Furthermore, breast cancer patients with a higher BMI were found to have a higher mortality risk. Participants with higher BMI than 40 kg/m^2^ were at a mortality risk greater than 2-fold when compared to participants with BMI from 18 to 24.9 kg/m^2^. This increased risk is associated with larger tumors, positive lymph node status, and the triple-negative tumor subtype.^36^, ^37^, ^38^, ^39^ Studies also showed that obesity is linked to the elevated risk of premenopausal estrogen receptor (ER)-negative breast cancer and triple-negative breast cancers.^40^^,^^41^ Interestingly, weight loss in adulthood, either after the age of 18 years or post-menopause, is linked with a decreased risk of postmenopausal breast cancer. This suggests that maintaining a healthy weight could reduce the risk of developing this disease.^42^^,^^43^ In the case of specific obesity-associated cancers, obesity is strongly linked to the development of ER-positive breast cancer in postmenopausal women.^44^ It triggers systemic changes like hyperglycemia and hyperinsulinemia and increases levels of adipose tissue-derived estrogens, adipokines, and inflammatory mediators (e.g., prostaglandin E2 [PGE2], tumor necrosis factor [TNF], and IL-6), stimulating cancer growth.^44^^,^^45^ These obesity-associated factors cause metabolic perturbations in tumor and non-neoplastic cells in the tumor microenvironment.^46^ For instance, insulin stimulates PI3-AKT signaling, leptin suppresses AMP-activated protein kinase (AMPK) signaling and stimulates hypoxia-inducible factor 1α (HIF1α) in hypoxic conditions in breast cancer cells.^47^^,^^48^ There is an overall shift towards aerobic glycolysis, an increase in glucose uptake, protein synthesis, and cell proliferation.^49^^,^^50^ Additionally, obesity induces chronic low-grade inflammation in white adipose tissue, which breast tissue is predominantly made out of. This facilitates interaction with cells and signals from adipose remodeled by obesity, leading to immune dysfunction, such as increased pro-inflammatory cytokine production, reduced T cell function, and alternative macrophage activation.^51^

### 2.2 Colorectal cancer

Colorectal cancer (CRC) ranks as the third most prevalent cancer globally, constituting around 10 % of all cancer cases. It also stands as the second leading cause of cancer-related deaths worldwide.^52^ Persistent obesity is strongly associated with colorectal cancer in both men and women. The risk of developing colorectal cancer increases by 18 % to 32 % in overweight and obese individuals.^53^ Obese individuals are also associated with worse colorectal cancer outcomes with higher recurrence and mortality rates.^54^ Obesity induces abnormal lipid metabolism, resulting in changes in adipokines and hormone levels and chronic low-grade inflammation.^55^ Obesity is associated with gut microbiota dysbiosis, disrupted bile acid homeostasis, and other metabolic abnormalities such as insulin resistance and hyperinsulinemia.^56^ All of these factors may play important roles in CRC tumorigenesis, specifically in the complex metabolic regulation and cell proliferation/growth of CRC cells.^57^

### 2.3 Ovarian cancer

Ovarian cancer (OC) is the cause of 2.5 % of all malignancies in women, with an estimated 12,740 deaths in 2024.^58^ Dysregulation of lipid synthesis and metabolism in OC has been observed, with low density lipoprotein (LDL) promoting proliferation in OC cell lines and a higher risk of cancer death. Statin use, which lowers cholesterol levels, is linked to reduced OC risk.^59^, ^60^, ^61^, ^62^, ^63^ Obesity, particularly in postmenopausal women, can elevate circulating estrogen and androgen levels, potentially contributing to OC pathogenesis.^64^, ^65^, ^66^, ^67^ Numerous meta-analysis and pooled-analysis studies showed a weak positive correlation between BMI and OC risk.^68^, ^69^, ^70^, ^71^ However, another study showed a 30 % higher risk of OC for obese individuals and a 16 % higher risk for overweight individuals compared to those of normal weight – but this link between obesity and OC is less significant compared to other cancers.^71^ Adult weight gain in postmenopausal women, particularly those with low hormone use, is associated with a 13 % higher risk of postmenopausal OC.^72^^,^^73^ Despite these findings, further studies are needed to explore the potential correlation between adiposity and premenopausal OC.

### 2.4 Prostate cancer

Prostate cancer is the second most common cancer in males worldwide, and it is the fifth leading cancer death. Prostate cancer caused an estimated 375,304 deaths in 2020.^74^ Obese prostate cancer patients undergoing active androgen deprivation therapy (ADT) face serious long-term side effects and weight/fat mass gain compared to non-obese patients.^75^, ^76^, ^77^, ^78^, ^79^, ^80^, ^81^, ^82^ Studies showed an 8–11 % higher risk of prostate cancer progression and mortality in obese men.^83^ Another study found a 15 % mortality risk increase for every extra 5 kg/m^2^ in BMI.^76^^,^^84^, ^85^, ^86^, ^87^ There are difficulties in detecting and treating obese prostate cancer patients with radical prostatectomy due to prostate enlargement and excess fat mass around the prostate (thus more distance between the skin surface to the prostate), leading to delayed diagnosis, disease progression, and longer surgery duration, blood loss as well as higher risk of capsular incision during surgery.^88^, ^89^, ^90^, ^91^, ^92^, ^93^

## 3 Molecular interconnections between obesity and cancer

### 3.1 Metabolic perturbations

Patients with obesity-associated cancers frequently exhibit a poorer prognosis, diminished treatment responses, and a heightened propensity for metastatic disease than their leaner counterparts. Several pathophysiological elements underpin the heightened cancer risk observed in obesity. Three primary contributors to this enhanced risk are metabolic perturbations, chronic inflammation, and growth factor imbalances.

A noteworthy manifestation of obesity is pancreatic steatosis, characterized by the accumulation of lipids in the pancreas. This, along with non-alcoholic fatty liver disease, significantly escalates inflammatory and metabolic disruptions. Intriguingly, pancreatic lipid accumulation has been identified as a potential risk factor for developing precancerous pancreatic lesions, underscoring its significance in cancer etiology.^46^^,^^94^

Interestingly, the *FTO* gene, a genetic key player in obesity, may provide a genetic link between these obesity-related metabolic disturbances and cancer, thereby offering a potential explanation for the observed association between obesity and tumorigenesis. While the *FTO* gene is particularly salient in obesity-related discussions, contemporary genome-wide association studies have elucidated its association with not just adiposity but also metabolic disturbances and, most concerningly, cancer.^15^ This gene's relevance to obesity was cemented by identifying single-nucleotide polymorphisms (SNPs) in individuals, both children and adults, diagnosed with obesity. Animal models, specifically the *FTO* knock-out mice, further corroborated this connection. When devoid of *FTO*, these mice exhibited significant body weight and fat mass reductions, specifically in white adipose tissue.^95^ Moreover, reduced FTO expression appears to have an intriguing biological effect. A distinct study demonstrated that when FTO levels decline, white adipocytes transform into their brown or beige counterparts, as evidenced by the amplified expression of the brown adipose tissue marker, UCP-1 protein.^96^ However, the sphere of influence of FTO isn't restricted only to adiposity. It also plays a pivotal role in the dopaminergic regulation of the midbrain by modulating D2R-D3R-GIRK-mediated signaling. Any disruption in this regulatory process can be a precursor to obesity.^97^ Mechanistically, certain mutations in the *FTO* gene heighten obesity risk. For instance, individuals possessing certain *FTO* risk alleles have shown predispositions to consume calorie-dense foods, which often culminate in overeating or uncontrolled eating behaviors.^98^ Several *FTO* gene variants, including rs9939609, rs8050136, rs1477196, rs6499640, rs1121980, rs17817449 and rs11075995 have been meticulously analyzed. Alarmingly, they've been consistently linked to increased cancer risks.^99^ Specifically, the *FTO* SNP rs9939609 has associations with many cancers, from lung and renal to more common types like breast and prostate.^100^ On a molecular level, FTO has been observed to diminish N6-methyladenosine levels, amplifying β-catenin expression. This modulation has concerning implications; it bolsters the chemo-radiotherapy resistance in cervical squamous cell carcinoma, as observed in both *in vitro* and *in vivo* models.^101^ It is plausible that these molecular changes could interact with the metabolic reprogramming seen in obese individuals, potentially exacerbating cancer cell proliferation and altering energy production pathways.

In individuals with obesity, a combination of hyperglycemia, dyslipidemia, and insulin resistance triggers metabolic reprogramming. This leads to an acceleration in the proliferation of cancer cells. Interestingly, during this process, the intermediates of the citric acid cycle are bypassed and not utilized for ATP production. This diversion deprives the mitochondria of nutrients while supplying the necessary precursors for lipid synthesis. Consequently, cancer cells voraciously uptake metabolized glucose, ensuring a steady supply of substrates to meet their proliferative demands. The facilitation of this process is evident in various cancer types, marked by an increased presence of glucose transporters and glycolytic enzymes.^46^^,^^102^^,^^103^ One of the key pathways implicated in this metabolic switch is driven by hyperinsulinemia. It activates the insulin growth factor pathway encompassing ligands such as insulin, insulin growth factor (IGF)-1, and IGF-2. These ligands interact with a variety of receptors, including insulin receptor (IR) α, IR β, IGF-1 receptor (IGF-1R), IGF-2R, and hybrid receptors like IGF-1R/IR α and IGF-1R/IR β. Moreover, these elements interact with IGF-binding proteins.^104^ Insulin, in particular, modulates the growth hormone, which in turn influences the production of IGF-1 and IGFBP-3. It's noteworthy that elevated levels of IGF-1 have been linked with increased risks of both pre and post-menopausal breast cancers, as well as prostate cancer.^105^ The role of sex hormones in the connection between obesity and cancer has also been highlighted. Specifically, estrogen can bind to its receptor, activating intracellular signaling pathways. This activation can fuel tumor progression by stimulating cell division. Furthermore, the interaction of estrogen with IGF can inhibit apoptosis, further promoting tumor growth.^106^

**Fig. 1:**
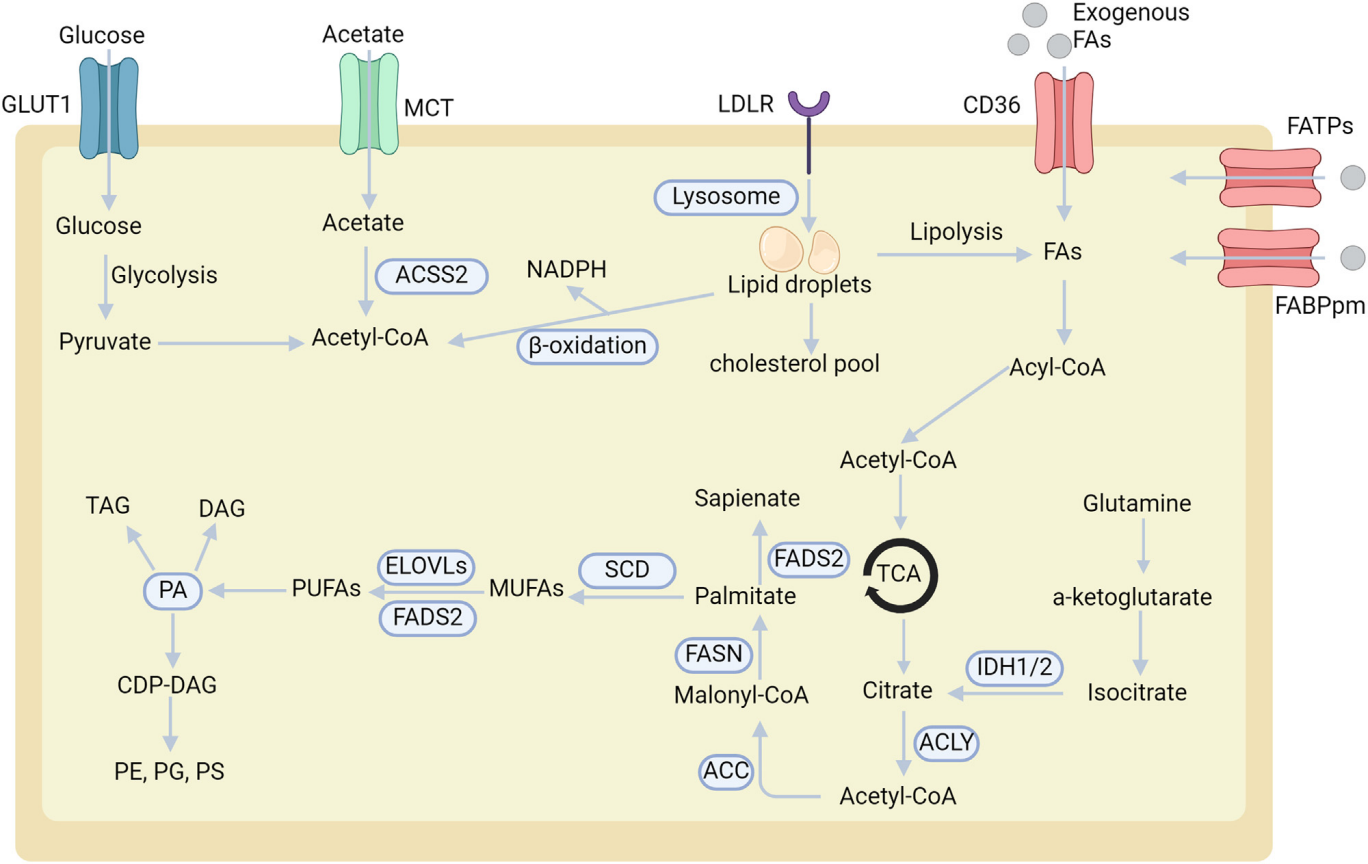
Overview of metabolic adaptations in cancer cells. Cancer cells exhibit distinct metabolic alterations to support their growth and survival. This illustration elucidates the key pathways and mechanisms involved. Cancer cells acquire FAs through *de novo* lipogenesis (where FAs are synthesized within the cell) and exogenous uptake (wherein FAs are absorbed from the tumor microenvironment, facilitated by transporters like CD36, FATPs, and FABPpm). Once inside, FAs are primarily stored in lipid droplets. These FAs can be metabolized to produce NADPH and acetyl-CoA via the β-oxidation process. Cancer cells metabolize glucose, glutamine, and acetate as primary substrates to produce citrate. Citrate is converted into palmitate through enzymatic reactions catalyzed by ACLY, ACC, and FASN. Subsequently, palmitate undergoes various modifications like desaturation and elongation, resulting in a diverse range of lipid species. Instead of the traditional pathway that leads to palmitoleate production, an alternative route exists where palmitate is desaturated to yield sapienate, primarily through the action of the FADS2 enzyme. ACC, acetyl-CoA carboxylase; ACSS2, acyl-CoA synthetase short-chain family member 2; ACLY, ATP–citrate lyase; CD36, cluster of differentiation 36; CDP, cytidine diphosphate; DAG, diacylglycerol; ELOVLs, elongation of very long-chain fatty acid protein; FAs, fatty acids; FABPpm, fatty acid-binding protein; FADS2, fatty acid desaturase 2; FASN, fatty acid synthase; FATPs, fatty acid transport proteins; GLS, glutaminase; GLUT1, glucose transporter 1; IDH, isocitrate dehydrogenase; LDLR, low density lipoprotein receptor; MCT, monocarboxylate transporter; MUFAs, monounsaturated fatty acids; NADPH, nicotinamide adenine dinucleotide phosphate; PA, phosphatidic acid; PE, phosphatidylethanolamine; PG, phosphatidylglycerol; PS, phosphatidylserine; PUFAs, polyunsaturated fatty acids; SCD, stearoyl-CoA desaturase-1; TAG, triacylglycerol.

Obesity often disrupts various lipid metabolic pathways, affecting fatty acid metabolism, cholesterol regulation, oxidation, and accumulation. These dysregulations act as catalysts, fostering the emergence of various malignancies. While many cells primarily rely on aerobic glycolysis for metabolism, cancer cells have adapted to extensively harness lipids and cholesterol to satisfy their continuous energy needs. The energy substrates, namely fatty acids, can either be sourced externally from the microenvironment or internally synthesized from abundant precursors like glucose or glutamine. Since obese individuals typically consume excessive calories, they possess an enlarged reservoir of glucose and glutamine, thereby facilitating the synthesis of additional fatty acids. The metabolic reprogramming observed in cancer cells involves shifts in fatty acid transport, *de novo* lipogenesis, lipid droplet storage, and β-oxidation. These lipidomic alterations have been recognized as one of the defining metabolic hallmarks of cancer.^107^ Certain cancers, such as diffuse large B-cell lymphoma,^122^ predominantly depend on lipids for their energy production.^108^ Cancer cells actively uptake exogenous lipids to satiate this need while boosting endogenous lipid synthesis. Free fatty acids, crucial for mitochondrial oxidation and energy production, are assimilated through transport mechanisms such as fatty acid translocase CD36, fatty acid transport proteins (FATPs)/SLC27A, and fatty acid-binding proteins (Fig. 1). On the other hand, cholesterol, which is crucial for forming membrane microdomains and initiating tumor growth, is primarily obtained through cholesterol-rich lipoproteins via low-density lipoprotein receptors (Fig. 1).^109^ Cholesterol is utilized to synthesize bile acids and steroid hormones, which can further exacerbate cancer progression. Studies have shown that many obese individuals have elevated serum levels of free fatty acids.^110^ These lipid elevations can lead to insulin resistance, inflammation, lipotoxicity, and endothelial dysfunction, primarily due to the consumption of highly saturated fats or uncontrolled lipolysis.^111^^,^^112^ These metabolic abnormalities, which are a result of obesity, significantly increase the risk of cancer development.

There is a well-established link between cholesterol levels and angiogenesis, a critical process in tumor growth. High levels of circulating cholesterol have been shown to promote tumor angiogenesis actively. On the other hand, cholesterol uptake inhibitors, such as ezetimibe, have proven effective in reducing tumor angiogenesis.^113^ In certain cancers, such as metastatic ovarian cancer, the transfer of fatty acids from adjacent adipocytes has been linked to impaired AMPK signaling. Oncogenic signaling pathways trigger changes in metabolic enzymes associated with lipid metabolism, significantly influencing the tumor lipidome. Another example is the activation of the PI3K–AKT pathway, one of the most frequently dysregulated signaling pathways in human cancers, via the stimulation of growth factor receptor tyrosine kinases, such as the human epidermal growth factor receptor 2 (HER2) and the IR.^114^ Notably, elevated HER2 levels in breast cancer have been intrinsically linked to the hyperactivation of the PI3 K signaling pathway.^115^^,^^116^

Moreover, several studies have observed increased circulating adipose fatty acid binding protein (AFABP) in obese mice and humans.^117^ In a study to uncover the source of elevated AFABP levels, serum samples were collected from obese women. These samples were taken before and at various intervals (3, 6, and 12 months) after undergoing Roux-en-Y gastric bypass surgery. The results of the study revealed a direct relationship between AFABP levels and BMI. As patients experienced changes in their BMI following the surgery, AFABP levels mirrored these changes, highlighting the intertwined relationship between them. This observed decrease in AFABP levels and a reduced BMI support previous data suggesting that AFABP primarily originates from adipose tissue in patients. Additionally, biopsies taken from obese women revealed significantly higher AFABP levels compared to those from lean individuals. This increased presence was consistently observed even among those with benign lesions. However, the rise in AFABP was especially noticeable in obese women diagnosed with breast cancer, reinforcing the hypothesis that an abnormal increase in AFABP plays a crucial role in the progression of breast cancer associated with obesity.^118^ The elevated AFABP level and its correlation with obesity, as discussed above, provide a crucial link to the development of ER-positive breast cancer in postmenopausal women.^44^ This interplay underscores the complex and multifaceted nature of cancer pathogenesis.

### 3.2 Cellular changes

Obesity-induced cellular changes intricately weave together metabolism and cellular growth dynamics. Non-malignant cells adjust their activity based on nutrient availability. Specifically, they engage in proliferation and anabolic processes when nutrients are plentiful. However, these cells shift to a non-proliferative catabolic state during nutrient scarcity, conserving energy and resources. AMPK plays a crucial role as a cell-signaling mediator, becoming activated under conditions of low nutrient availability. The primary trigger for AMPK activation is increased AMP levels, which signals an energy deficit. In the context of obesity-related cancers, adiponectin levels noticeably decrease. This reduction in adiponectin subsequently stimulates AMPK. Once activated, AMPK serves as a metabolic checkpoint, inhibiting the mTOR pathway, promoting a catabolic state, and suppressing cellular proliferation.

On the other hand, the AKT pathway, activated by insulin when nutrients are abundant, plays a key role in regulating lipid and glucose metabolism within cells. This kinase primarily influences cellular functions by stimulating the mTOR pathway, which prevents apoptosis and drives cellular proliferation. Interestingly, AMPK and AKT serve as counterbalances in cellular metabolism. While AKT fosters growth and proliferation in nutrient-rich environments, AMPK strives to maintain energy homeostasis, initiating energy-conserving mechanisms during nutrient scarcity.

PTEN acts as a vital intermediary between the AMPK and AKT pathways. It inhibits the activation of AKT and stimulates AMPK, particularly in response to nutrient depletion. This dual action of PTEN fosters a catabolic state in cells and inhibits cellular proliferation, primarily by influencing the downstream mTOR pathway.^119^

Increased body weight exerts profound cellular effects, particularly in relation to oxidative stress and metabolic changes, both of which significantly contribute to cancer development and progression. Increased body weight is linked to a surge in reactive oxygen species (ROS) production. At high concentrations, these molecules can stimulate tumor initiation, accelerate its progression, and enhance the vascularization processes that support tumor growth.^120^ High levels of adipose tissue can trigger proinflammatory cascades, promoting lipolysis and releasing free fatty acids. The accumulation of these free fatty acids can lead to many problems, including lipotoxicity, insulin resistance, inflammation, and more comprehensive metabolic disturbances.^121^ The accumulation of free fatty acids in the liver can also induce insulin resistance, increase hepatic glucose production, stimulate inflammation, and disrupt hepatic triglyceride and cholesterol metabolism, all contributing to dyslipidemia.^19^ If the body cannot counteract insulin resistance by producing more insulin, this can result in hyperglycemia and eventually type 2 diabetes mellitus. Hyperglycemic states enhance mitochondrial glucose metabolism, leading to further mitochondrial dysfunction, increased ROS production, and the conversion of glucose to sorbitol.^122^ A study highlighted the induction of oxidative stress due to acute hyperglycemia, which increased circulating levels of inflammatory cytokines, including IL-6, IL-18, and TNF-α.^123^ These inflammatory markers play pivotal roles in the development and progression of tumors.^124^ Resistin, an adipose-derived hormone discovered in 2001, was named for its role in conferring resistance to insulin. It was identified as a novel factor secreted by adipocytes that impaired insulin functions in obese mice.^125^

Adipokines, key molecules secreted by adipose tissue, play crucial roles in various metabolic processes. Their dysregulation is related to obesity-driven carcinogenesis. Beyond the well-established adipokines like adiponectin and leptin, resistin and visfatin have also come to the forefront in recent studies on obesity and cancer. Adiponectin's levels are inversely proportional to the prevalence of endometrial, breast, colon, and prostate cancers.^126^ Adiponectin exhibits anti-inflammatory properties that influence inflammation associated with obesity and cancer.^106^

Leptin induces the expression of pro-inflammatory and pro-tumorigenic cytokines, such as IL-1, IL-6, and TNF-α within macrophages.^127^ These cytokines contribute to the onset and progression of breast tumors in obese women.^16^^,^^128^

Resistin, another adipokine, has been associated with metabolic disorders, including insulin resistance, type 2 diabetes, and atherosclerotic cardiovascular disease.^129^ A characteristic feature of obesity is the alteration in lipid levels, which is linked to increased resistin levels.^130^ A study from Tunisia underscored significantly higher resistin levels in obese individuals compared to their non-obese counterparts.^131^ Resistin is also implicated in inflammation and endothelial activation, highlighting its role in obesity-induced cancer.^130^ Numerous studies have reported elevated resistin levels in both premenopausal and post-menopausal breast cancer patients, suggesting its potential as a diagnostic marker for breast cancer.^132^, ^133^, ^134^ Notably, breast cancer patients with a BMI exceeding 25kg/m^2^ displayed higher serum resistin levels than controls with a BMI below this threshold.^134^^,^^135^ Given the excessive accumulation of visceral adipose tissue observed in breast cancer,^136^^,^^137^ a clear link exists between resistin and obesity-induced carcinogenesis.

Intracellular and extracellular visfatin has been associated with cancer development by promoting cellular growth, angiogenesis, and metastasis through the MAPK, AKT, STAT3, and NF-κB/Notch1 pathways in macrophages, human endothelial cells, and breast cancer cells (Table 1). Furthermore, visfatin triggers the GDF15-AKT mechanism in adipose-derived stem cells, promoting breast cancer progression.^138^^,^^139^ Elevated circulating visfatin levels have been observed in patients with endometrial cancer and hepatocellular carcinoma, suggesting its wider implications in obesity-associated malignancies.^126^^,^^140^, ^141^, ^142^

**Table 1:** Adipokines-induced signaling pathways in different cancer cell lines.

Type of cancer	Mechanism			
	Leptin	Adiponectin	Resistin	References
Breast cancer	Increases proliferation through activation of SREBP-1. Increases metastasis through upregulation of plasminogen activator inhibitor-1 by leptin-LEPR-miR-34a axis-mediated STAT3 signaling activation	Decreased levels of adiponectin and elevated levels of IL-1, IL-6, and TNF-α leads to the activation of MAPK and LKB1/AMPK signaling inhibition, leading to cell growth, proliferation, survival, and anti-apoptotic response.	Upregulates pro-survival signals, such as BCL-2 and BCL-Xl, and downregulates pro-apoptotic signals, such as cleaved caspases and PARP. It also upregulates the expression of mesenchymal markers, such as vimentin, ZEB1, N-cadherin, SNAIL, SLUG, TWIST1, and fibronectin. Induces metastasis through CAP1, NF-κB, STAT3, and intracellular calcium concentration-dependent Src/PP2A/ PKCα signaling pathways.	230, 231, 232, 233, 234, 235
Colorectal cancer	Increases proliferation through activation of STAT3 signaling. Increases metastasis through PI-3 K, Rho- and Rac-dependent pathways	Decreases the PI3K/Akt signal pathway activation induced by leptin. Regulates mTOR, the cell cycle and cell growth Fas/Fas ligand, NF-κB, Bcl-xl and p53 in cell survival; glycogen synthase kinase, p27, Rac and cdc42 in the cell cycle; mTOR in cell growth. Src for cytoskeleton modification for cancer cell metastasis. It also inhibits inflammatory factors such as IL6 and TNFα. IL6 and TNFα are found in high levels of obesity and activate the PI3K/Akt pathway.	Regulates matrix metalloproteinases production and modulates VEGF secretion, which are key factors in promoting tumor invasion.	^207^^,^^236^, ^237^, ^238^, ^239^, ^240^, ^241^, ^242^
Prostate cancer	Increases proliferation and decreases apoptosis through ERK1/2 signaling activation. Increases metastasis through activation of STAT3	Adiponectin regulates the AMPK signaling cascade to its receptor. The proteins downstream of AMPK included TSC2, mTOR, VEGF-A, and fatty acid synthase, which all regulate cell proliferation. Adiponectin activates AMPK in PC-3 cells, lowering mTOR activation, reducing protein translation, and inhibiting cell growth.	Promotes phosphorylation of Src, which is a downstream target of FAK kinase and leads to high levels of intracellular calcium. FAK/Src pathway induces Akt activation in cancer cells, leading to high secretion of MMP-2 and MMP-9, which eventually causes proliferation, migration, invasion, and metastasis.	243, 244, 245, 246, 247
Pancreatic cancer	Increases proliferation and drug resistance to gemcitabine/5-Fluorouracil by increasing cellular glucose uptake	Decreases proliferation significantly by attenuating the β-catenin pathway but does not affect migration and invasion	No study so far	248, 249, 250, 251
Lung cancer	Increases proliferation, metastasis, and drug resistance to erlotinib/cisplatin through the ERK1/2 pathway.	Inhibits the proliferation of human lung adenocarcinoma and decreases the cell division cycle. It also has an anti-proliferative effect by decreasing CREB activation. CREB increases the survival, growth, and differentiation of cancer cells	Increases PI3 K, p85 and Akt phosphorylation via the TLR4/Src/EGFR pathway. Repression of nuclear translocation of NF‐κB through TLR4, Src, EGFR, and PI3 K inhibitors. Regulation of MMP2 and Twist1 expression by NF-κB. Resistin leads to lung adenocarcinoma cell migration and invasion through the TLR4/Src/EGFR/PI3K/NF‐κB pathway.	^237^^,^^252^, ^253^, ^254^, ^255^, ^256^
Brain tumor	Increases proliferation, metastasis, angiogenesis, and drug resistance to temozolomide through Src/ERK/Akt/mTOR/p70S6K/rS6 pathway	No study so far	No study so far	257, 258, 259
Ovarian cancer	Increases proliferation, metastasis, and drug resistance to paclitaxel/docetaxel through the ERK1/2 pathway	Promotes chemokine ligand 1 secretion in ovarian cancer, which leads to VEGF-independent angiogenesis. It also decreases cell proliferation in epithelial ovarian cancer.	Affects the PI3K–Akt–Sp1 pathway via increasing Sp1 interaction, leading to progressive phosphorylation of Sp1 on Thr453 and Thr739 which leads to angiogenesis	^237^^,^^260^^,^^261^
Gall bladder cancer	Increases proliferation through the activation of SOCS3/JAK2/p-STAT3 pathway	No study so far	No study so far	^262^
Liver cancer	Increases proliferation, metastasis and decreases apoptosis through activation of STAT3 and ERK1/2	Modulates JNK, AMPK and mTOR signaling pathways by stimulating the phosphorylation of JNK and AMPK and decreasing activation of mTOR. These lead to anti-proliferative and pro-apoptotic activities in hepatocellular carcinoma cells.	Increases invasion via both the PI3 K and ERK signaling pathways.	263, 264, 265, 266

Abbreviation: Sp1, specificity protein 1.

Moreover, apelin, an adipokine, regulates blood pressure regulation and energy metabolism and has been linked to the pathological processes of obesity and cancer. The apelin levels are interconnected with the plasma insulin level, with insulin administration to obese mice resulting in increased apelin transcription and activation of the PI3K/Akt, PKC, and MAPK pathways.^143^ Moreover, its function in angiogenesis through enhancing lymphatic and blood vessel integrity and its role in cell migration can stimulate tumor growth and proliferation (Table 1).^144^ Salman et al. found increased serum apelin-36 levels in postmenopausal breast cancer patients compared to normal individuals, and this was positively correlated with BMI.^145^

A closer look at the cellular changes reveals that the intricate relationship between obesity and cancer is not merely about excess fat or high BMI. Instead, it is fundamentally anchored in the molecules that serve as intermediaries between them. Once believed to perform metabolic functions solely, these molecules have revealed themselves as critical players in carcinogenesis. As researchers, we aim to unravel these connections and harness this knowledge to enhance human healthcare. The study of resistin and visfatin offers not just deeper insights into the intricate interplay between obesity and cancer but also serves as a reminder that every molecule in our body harbors a story waiting to be told—a story that could potentially revolutionize medicine.

### 3.3 Inflammatory changes

**Fig. 2:**
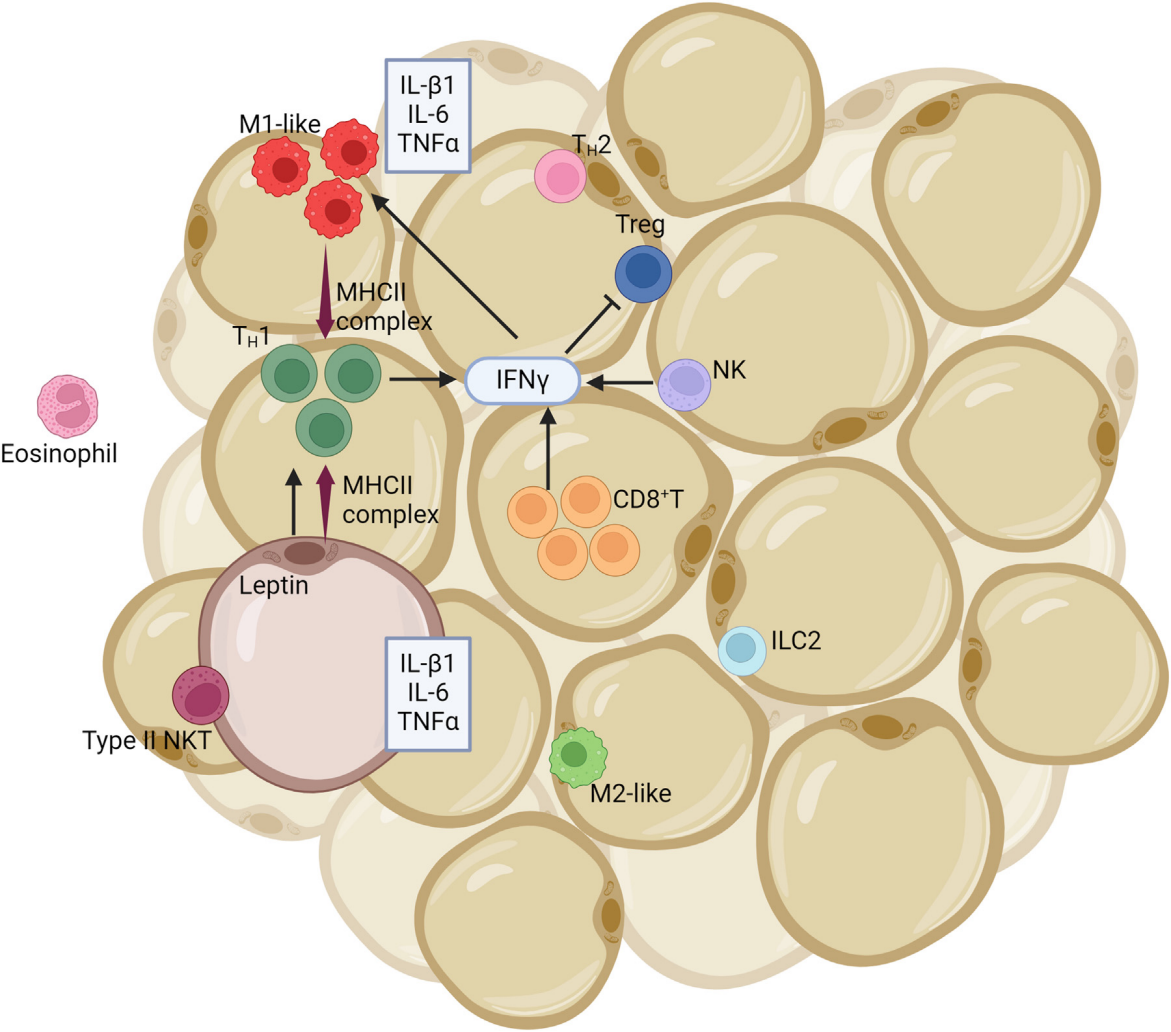
Inflammation-induced alterations in adipose tissue dynamics. This illustration captures the complex interplay between adipose tissue and immune cells during inflammation, particularly in the context of obesity. Obese-state adipocytes show elevated leptin expression, a hormone involved in appetite regulation and inflammation. Concurrently, these adipocytes also express II MHCII, which plays a role in immune cell recognition. MHCII-expressing adipocytes, in the presence of myeloid cells, facilitate the activation of T_H_1 cells. Upon T_H_1 activation, there's a subsequent secretion of the cytokine IFNγ. This cytokine is not just released by T_H_1 cells but also by NK and CD8^+^ T cells. The released IFNγ promotes further expression of adipocyte MHCII and encourages the polarization of macrophages towards the pro-inflammatory M1 phenotype. The aforementioned processes coincide with reduced abundance and function of Treg and T_H_2 cells. Both these cell types generally exert anti-inflammatory effects. Adipocytes, along with M1-polarized macrophages, release pro-inflammatory cytokines, specifically IL-1β, IL-6, and TNFα. These cytokines amplify the inflammatory cascade within the adipose tissue. Collectively, these mechanisms perpetuate inflammation in adipose tissue and are marked by reduced levels of Treg and T_H_2 cells, further compromising the tissue's anti-inflammatory response. IFNγ, interferon γ; IL, interleukin; ILC2, innate lymphoid type 2 cell; MHCII, class II major histocompatibility complex; NK, natural killer; NKT, natural killer T; T_H_1, T helper type 1; T_H_2, T helper type 2; TNFα, tumor necrosis factor α; Treg, regulatory T cell.

**Fig. 3:**
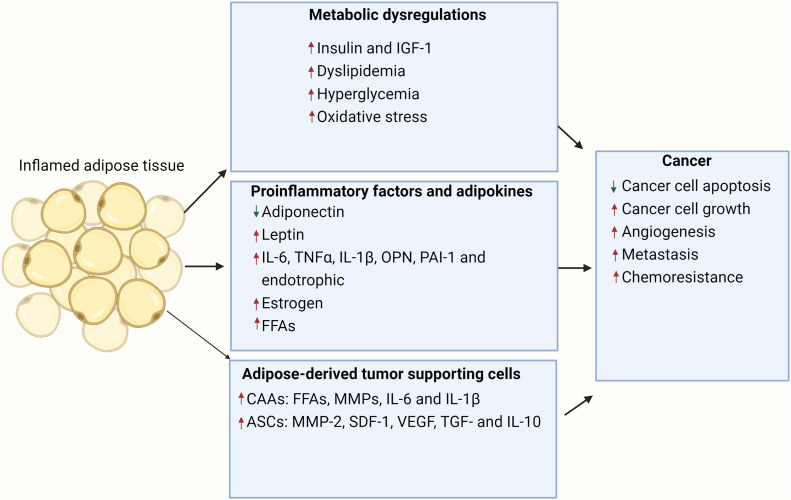
The multi-faceted link between obesity and cancer development. This illustration delves into the intricate mechanisms by which expanded inflamed adipose tissue during obesity can accentuate cancer progression. Obesity is characterized by a significant increase in the size and inflammation of adipose tissue, contributing to a host of downstream effects. Inflamed adipose tissue releases adipokines, which are bioactive molecules. These adipokines have been implicated in enhancing tumor growth, metastasis and causing metabolic disturbances. Adipose tissue gives rise to CAAs, which in turn release FFAs and various inflammatory cytokines. These factors can directly influence cancer cell behavior and proliferation. ASCs, derived from adipose tissue, play a pivotal role in remodeling the tumor microenvironment. Their interaction with the surrounding matrix and cells can facilitate a niche conducive to tumor growth. Beyond direct interactions with tumors, adipose-derived proinflammatory factors have systemic effects. They can modify global metabolism and alter the behavior of tumor-supporting cells, fostering an environment favorable for tumor expansion. Collectively, these processes detail how obesity, through inflamed adipose tissue, can generate a milieu that is both directly and indirectly conducive to cancer development and progression.^19^ ASCs, adipose-derived stem cells; CAAs, cancer-associated adipocytes; FFAs, free fatty acids; IGF-1, insulin growth factor-1; IL, interleukin; MMP, matrix metalloproteinase; OPN, osteopontin; PAI-1, plasminogen activator inhibitor-1; SDF-1, stromal-derived factor-1; TGF-β, transforming growth factor β; TNFα, tumor necrosis factor α; VEGF, vascular endothelial growth factor.

The intricate relationship between obesity and inflammation, underscored by cellular and metabolic changes, is increasingly recognized as a precursor to cancer. The cascade of events, from the mere accumulation of excess nutrients to the emergence of inflammation, illustrates the delicate equilibrium our bodies strive to maintain. Obesity lays the groundwork for inflammation. Key metabolic tissues like the liver, pancreas, brain, and, crucially, white adipose tissue become the primary battlegrounds for obesity-induced inflammation.^146^ The influx of excess nutrients activates metabolic signaling pathways – c-Jun N-terminal kinase, NF-κB, and protein kinase R, leading to a surge in low-level inflammatory cytokines and instigating a mild inflammatory response.^147^^,^^148^ Amidst this inflammatory response triggered by obesity, white adipose tissue plays a pivotal role, a unique tissue type with distinct characteristics and functions. White adipose tissue, characterized by its white or yellow color and less vascularization and innervation than brown tissue, produces a vast array of adipokines and lipokines.^149^ They also produce proteins with a wide range of functions related to proinflammatory cytokines, immunity, and lipid metabolism.^150^ Lipokines, lipids produced by adipocytes, play a role in metabolism.^151^ Brown adipose tissue, unlike white adipose tissue, originates from muscle tissue precursor cells,^152^ its brown color is due to its vascularization and abundant mitochondria containing cytochromes. The fat cells within brown adipose tissue are multilocular, containing many lipid vacuoles. Unlike white adipose tissues, brown adipose tissue does not store energy but uses it through thermogenesis.^149^ Beige adipose tissues, which share features with brown adipose tissues, such as having many lipid vacuoles, develop from different embryonic precursors.^153^ When obesity burdens white adipose tissue, significant changes occur. There's a noticeable growth in the size (hypertrophy) and number (hyperplasia) of adipocytes, leading to a shift in adipokine production. This shift, coupled with the increased free fatty acids, drives a subtle yet persistent inflammatory reaction (Fig. 2).^154^ Obesity also escalates endoplasmic reticulum stress, triggering the unfolded protein response. This cascade intensifies the activation of c-Jun N-terminal kinase, NF-κB, and heightens oxidative stress, increasing inflammatory cytokines.^155^ Additionally, obesity induces a higher death rate among adipocytes, which subsequently release more free fatty acids. These fatty acids serve as signals, attracting macrophages to clear the cellular debris in white adipose tissue. Dead adipocytes and specific chemokines amplify this macrophage recruitment, including chemokine ligands 2, 3, and RANTES/chemokine ligand 5 (Fig. 3).^156^ These mechanisms result in high levels of adipose tissue macrophages in obese white adipose tissue. White adipose tissue triggers a shift in the polarization of macrophages, from an anti-inflammatory M2-like phenotype to a pro-inflammatory M1-like phenotype in obese white adipose tissue due to the change in leptin and adiponectin levels (Fig. 3).^157^ Recent research sheds light on the involvement of breast adipose tissue in the drama of obesity-driven inflammation. Here, too, the familiar tale of adipocyte proliferation, cell death, cytokine outpouring, and macrophage invasion plays out.^158^

A deep exploration into the genetic landscape reveals that transcriptomic analyses of human ER positive breast cancer samples from obese patients exhibit a stark contrast in gene expression profiles compared to their non-obese counterparts. Particularly striking was the prominence of genes associated with inflammation and immune cell migration, suggesting that obesity remodels the genetic terrain of the tumor microenvironment.^159^ A fascinating interaction is observed between adipocytes and breast cancer cells. Their mutual interaction amplifies the production of inflammatory cytokines, thereby enhancing the aggressive nature of cancer cells and their tumor-forming potential.^160^ Leptin, which is notoriously abundant in obesity, triggers the expression of a host of pro-inflammatory and pro-tumorigenic cytokines, notably IL-1, IL-6, and TNF-α within macrophages.^127^ Experiments in rats have intriguingly highlighted the concurrent rise of IL-1β with leptin amidst increased body mass, propelling growth in the mammary epithelium.^161^ Alarmingly, these surges in cytokines are correlated with escalated breast cancer growth rates and unfavorable patient prognoses, underscoring their abundance in obese white adipose tissue.^162^ Further analysis of breast cancer patient samples reinforces this point, revealing an increased presence of crown-like structures in white adipose tissue, which is concurrently linked with heightened tumor proliferation, growth, and inflammatory cytokine production.^163^ However, leptin's role extends beyond cytokine modulation. Intriguingly, it also acts as a potent stimulator of aromatase, a crucial enzyme in the estrogen production pathway.^163^ An examination of the breast white adipose tissue of breast cancer patients unveils a narrative of inflammatory turmoil. Cytokines like TNF-α, IL-1β, and IL-6 not only fuel tumor growth in obesity-afflicted mouse models but also parallelly increase in obese women, closely correlating with the onset and progression of breast tumors.^16^^,^^128^

### 3.4 Tumor microenvironment dynamics

The tumor microenvironment (TME) serves as a sophisticated ecosystem for the tumor, ensuring its survival, growth, and potential spread. This intricate environment comprises a diverse range of components, such as immune cells, blood vessels, fibroblasts, lymphocytes, extracellular matrix, and various signaling molecules.^164^, ^165^, ^166^ The TME is a dynamic battleground for both malignant and non-malignant cells, undergoing significant changes to meet the needs of growing tumor cells. Contrary to what one might expect, non-malignant cells within the TME do not mean benign outcomes. It can have sinister implications, fostering uncontrolled cell proliferation throughout the various stages of cancer development.^167^^,^^168^ Malignant cells thrive in this nurturing environment and can invade distant healthy tissues using the lymphatic or circulatory systems. The immune system's role in the TME is multi-faceted, displaying protective and detrimental actions. Protective immune responses involve NK cells, which induce apoptosis in tumor cells using perforins and granzyme.^169^ B cells contribute by manufacturing antibodies targeted explicitly against tumor cells, while T-cells, particularly CD4 and CD8 subsets, orchestrate an anti-tumor response by interacting with cell surface markers.^170^ Conversely, the TME can also suppress beneficial immune reactions. For instance, M2 macrophages release transforming growth factor (TGF)-beta, dampening NK cell activity. Regulatory cells, such as B-regs and T-regs, counteract the effects of B-cells and T-cells, respectively, muffling the desired anti-tumor response.^169^ In the context of obesity, there's a pronounced influence on TME dynamics. Adipocytes, manipulated by tumor cells, transform to acquire a cancer-associated phenotype. This alteration leads to an increased adipose presence around tumors, further escalating the levels of pro-tumor cytokines like IL-6 and IL-1β.^171^ These cytokines facilitate the release of energy-rich free fatty acids from adipocytes, fueling the voracious metabolic demands of rapidly growing tumors.^172^

**Fig. 4:**
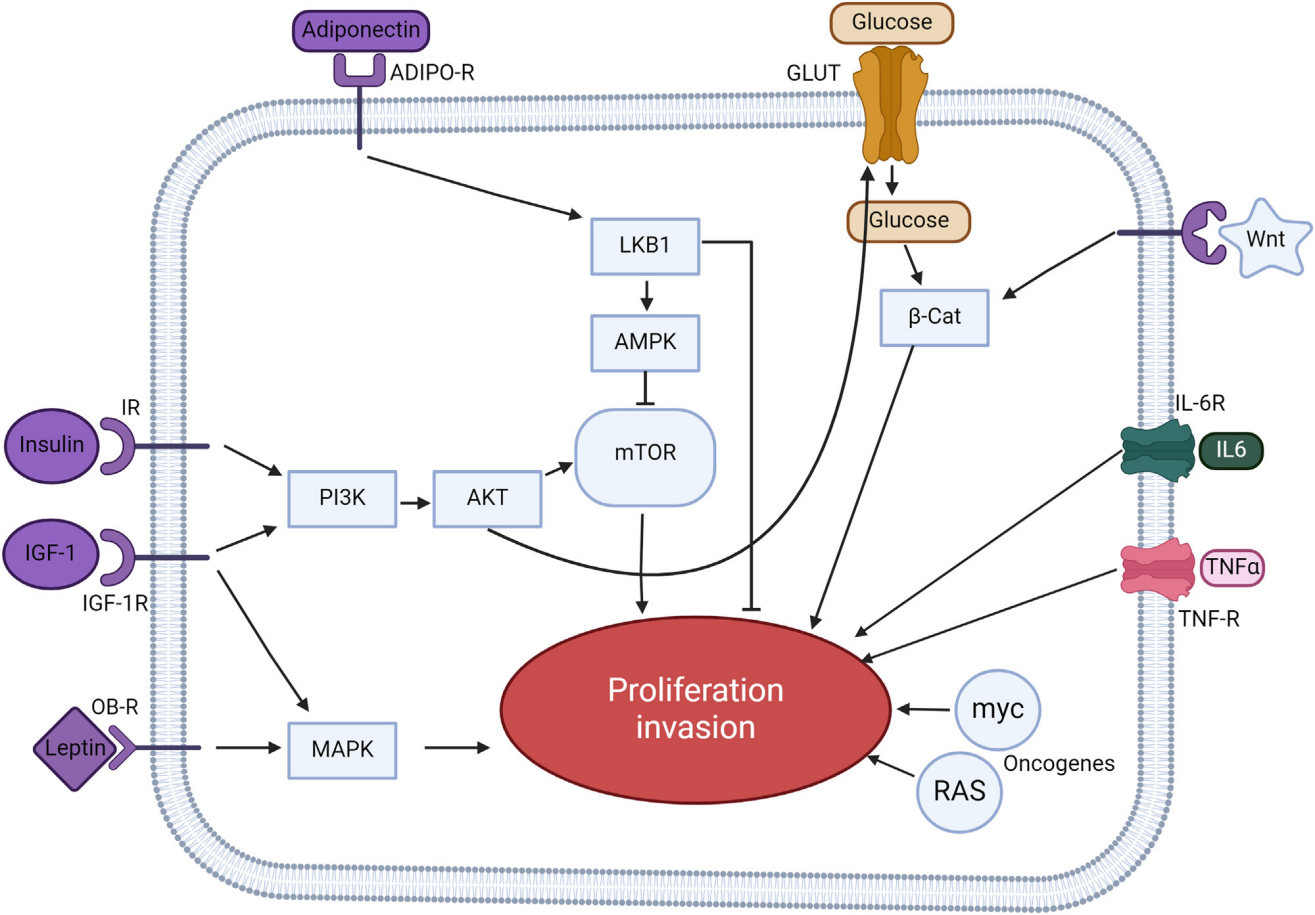
Intricate signaling pathways linking obesity to cancer progression. This figure provides a detailed account of the multiple signaling pathways implicated in connecting obesity to cancer. Insulin and IGF-1, when bound to their respective receptors (IR and IGF-1R), trigger the PI3K/AKT/mTOR signaling pathway. This cascade promotes tumor cell proliferation and invasion. Adiponectin, a hormone released predominantly by adipose tissue, interacts with ADIPO-R1 and ADIPO-R2. This binding initiates the LKB1/AMPK pathway which subsequently inhibits the mTOR pathway, acting as a counter-mechanism against tumor proliferation and metastasis. The insulin-driven PI3K/AKT pathway facilitates glucose uptake into cells via glucose transporters. Elevated glucose concentrations then amplify the Wnt/β-catenin signaling, further propelling tumor cell proliferation and invasion. Leptin, another hormone from adipose tissue, upon binding to its receptor OB-R, instigates the MAPK pathway. This cascade enhances cellular proliferation, augmenting tumor growth. Inflammatory cytokines IL-6 and TNFα, once bound to their receptors (IL-6R and TNF-R), activate the JAK/STAT/NF-kB signaling pathway. This cascade not only shields cells from apoptosis but also accentuates proliferation and metastasis. RAS and myc, being oncogenic proteins, modulate the expression of various metabolic enzymes. Their actions ramp up glycolysis, providing an energetic and metabolic advantage to tumor cells, leading to accelerated proliferation. ADIPO-R, adiponectin receptor; β-Cat, β-catenin; IGF-1, insulin growth factor; IGF-1R, insulin growth factor-1 receptor; IL6, interleukin 6; IL-6R, interleukin 6 receptor; IR, insulin reception; OB-R, leptin receptor; TNFα, tumor necrosis factor α; TNF-R, tumor necrosis factor receptor.

Additionally, adipose-derived stem cells release agents like matrix metalloproteinase-2 and stromal-derived factor-1, which enhance tumor invasion.^19^ Elevated insulin and IGF1 levels, particularly evident in obese states, profoundly affect the TME. These factors activate the PI3K/AKT/mTOR pathway upon binding to their respective receptors, promoting tumor proliferation and invasiveness (Fig. 4). Given the clear connection between obesity and altered TME dynamics, a deeper dive into the molecular mechanisms is warranted. The intricate relationship between obesity, the TME, and cancer progression offers numerous avenues for exploration, each with the potential to revolutionize our understanding and treatment of cancer in the modern age.

### 3.5 Extracellular vesicles as mediators in the obesity-driven cancer progression

Extracellular vesicles (EVs) are small, membrane-bound particles secreted by cells into the extracellular environment. These vesicles play a pivotal role in cell-to-cell communication by transporting bioactive molecules such as proteins, lipids, and nucleic acids. EVs are classified into three main subtypes based on their size and biogenesis pathways: exosomes (ranging from 70 to 150 nm), microvesicles (100 to 1000 nm), and apoptotic bodies (500 nm to several micrometers).^173^ Exosomes are formed within the endosomal system and released into the extracellular space upon fusion of multivesicular bodies with the plasma membrane. In contrast, microvesicles bud directly from the plasma membrane, and apoptotic bodies are formed during the process of programmed cell death. Obesity is characterized by chronic low-grade inflammation, insulin resistance, and metabolic dysregulation, all of which contribute to an increased risk of developing various cancers. Recent studies have highlighted the role of EVs in mediating obesity-related metabolic perturbations and their subsequent impact on cancer progression.^174^ The cargo of EVs, including proteins, lipids, and miRNAs, is altered in the context of obesity, and these changes are implicated in the modulation of key cellular processes such as differentiation, proliferation, apoptosis, and tumorigenesis.^174^, ^175^, ^176^ Several *in vitro* and *in vivo* studies have demonstrated that EVs derived from obese individuals or adipocytes significantly enhance cancer progression. For instance, EVs from the adipose tissue of obese individuals have been shown to carry pro-tumoral molecules that increase the malignancy of cancer cells. These vesicles promote cell migration, invasion, and resistance to apoptosis, which are critical processes in cancer metastasis.^177^^,^^178^ Notably, exosomes from obese adipocytes have been found to enhance the progression of breast cancer by influencing the tumor microenvironment and promoting a more aggressive phenotype. One particularly compelling study examined the effects of exosomes isolated from the plasma of obese women on the MCF-7 breast cancer cell line. The results demonstrated that these exosomes significantly increased the viability, migration, invasion, and tamoxifen resistance of the cancer cells compared to exosomes obtained from normal-weight women.^179^ This finding suggests that the obesity-related alterations in exosome content may contribute to the development of drug resistance in breast cancer, highlighting a potential mechanism by which obesity exacerbates cancer outcomes. Triple-negative breast cancer (TNBC) is an aggressive subtype of breast cancer that lacks targeted therapies. Recent studies have explored the influence of EVs on TNBC, particularly in the context of obesity. A study investigating the effects of EVs from women with varying BMI and metabolic health statuses found that EVs from obese individuals—whether metabolically healthy or unhealthy—significantly increased the invasion and migration of MDA-MB-231 TNBC cells.^180^ Additionally, these EVs were associated with increased matrix metalloproteinase-2 (MMP-2) activity, which plays a crucial role in the degradation of the extracellular matrix, facilitating cancer cell invasion. The study also revealed that EVs from metabolically unhealthy obese women exhibited decreased levels of p53 and phosphorylated p53, key regulators of cell cycle arrest and apoptosis. This reduction in p53 signaling was associated with decreased apoptosis in TNBC cells, further promoting cancer cell survival and aggressiveness. These findings suggest that the metabolic health of individuals, in conjunction with BMI, plays a critical role in shaping the impact of EVs on cancer behavior. In another study, EVs derived from patients with obesity hypoventilation syndrome were found to enhance the aggressiveness of lung adenocarcinoma cells.^181^ These EVs promoted cancer cell proliferation, migration, and invasion, further illustrating the role of obesity-associated EVs in driving cancer progression across different cancer types.

The role of EVs in the obesity-cancer link involves complex mechanisms that are still being unraveled. One proposed mechanism is the transfer of oncogenic miRNAs via EVs, which can modulate gene expression in recipient cells, leading to altered cellular behavior. For example, certain miRNAs carried by EVs from obese individuals have been shown to target tumor suppressor genes, thereby promoting tumor growth and metastasis. Additionally, EVs can modulate the immune response by carrying immunosuppressive molecules that facilitate tumor immune evasion, further contributing to cancer progression. Given their significant role in mediating the obesity-cancer link, EVs represent a promising target for therapeutic intervention. Strategies aimed at inhibiting the release of EVs, altering their cargo, or blocking their uptake by recipient cells could potentially mitigate the pro-tumoral effects of obesity-associated EVs. Moreover, EVs themselves could be harnessed as delivery vehicles for therapeutic agents, given their natural ability to target specific cells and tissues.

In summary, EVs are emerging as key players in the link between obesity and cancer. The alterations in EV cargo associated with obesity contribute to various oncogenic processes, including enhanced cancer cell migration, invasion, and drug resistance. The impact of EVs on cancer is influenced by both BMI and metabolic health, underscoring the complexity of the obesity-cancer relationship. Future research should continue to explore the mechanistic underpinnings of EV-mediated cancer progression and investigate the potential of EV-targeted therapies in combating obesity-related cancers.

## 4 Gut microbiome and obesity-associated cancer

Obesity and cancer, both multi-faceted conditions, share underlying ties to nutrient and energy intake, characterized by a constellation of low-grade inflammation, adiposity, and insulin resistance. The significance of gut microbiota in this equation cannot be understated. When mice were transplanted with the microbiota from obese individuals, they exhibited heightened adiposity and body mass compared to their counterparts transplanted with microbiota from lean individuals.^182^ Such shifts in microbiota, termed dysbiosis, can disrupt the natural microbial equilibrium, inciting systemic inflammation. This inflammation cascade is often triggered by gut microbiota molecules, particularly lipopolysaccharides (LPS), leading to endotoxemia. Further complicating the milieu, the absence of specific microbes disrupts gut lymphoid tissue integrity, while others, like *Bacteroides fragilis,* play a protective role by rectifying host T-cell deficiencies.^183^^,^^184^ Obesity and gastrointestinal cancer are intertwined through these inflammatory pathways. Certain microbes bear associations with gastrointestinal cancer development, with intricate signaling pathways, such as NF-κB, TLR4, and NOD, playing pivotal roles. This microbial-host dialogue involves signaling molecules like flagellin, peptidoglycans, and, notably, short-chain fatty acids (SCFA). SCFAs, products of bacterial fermentation, are paramount in colon health. Specifically, butyrate aids in epithelial cell renewal in the intestine, orchestrating gene expressions pivotal for this renewal.^185^^,^^186^ Moreover, the intricate relationship between diet and the gut is exemplified by the gram-negative bacterium *Akkermansia muciniphila*. Its presence mitigates diet-induced gut barrier dysfunctions in mice.^186^^,^^187^

The development of CRC is intricately associated with the gut's microbial environment. Several bacterial species, notably *Escherichia coli, Fusobacterium nucleatum, Streptococcus bovis, Helicobacter pylori, Bacteroides fragilis, Enterococcus faecalis*, and *Clostridium septicum*, have been identified as key players in CRC's onset and progression.^188^^,^^189^ Gut microbiome dysbiosis, or the imbalance in the microbial community, predicts obesity-associated CRC, irrespective of BMI.^190^ This suggests that factors beyond mere weight play critical roles in CRC pathogenesis.

**Fig. 5:**
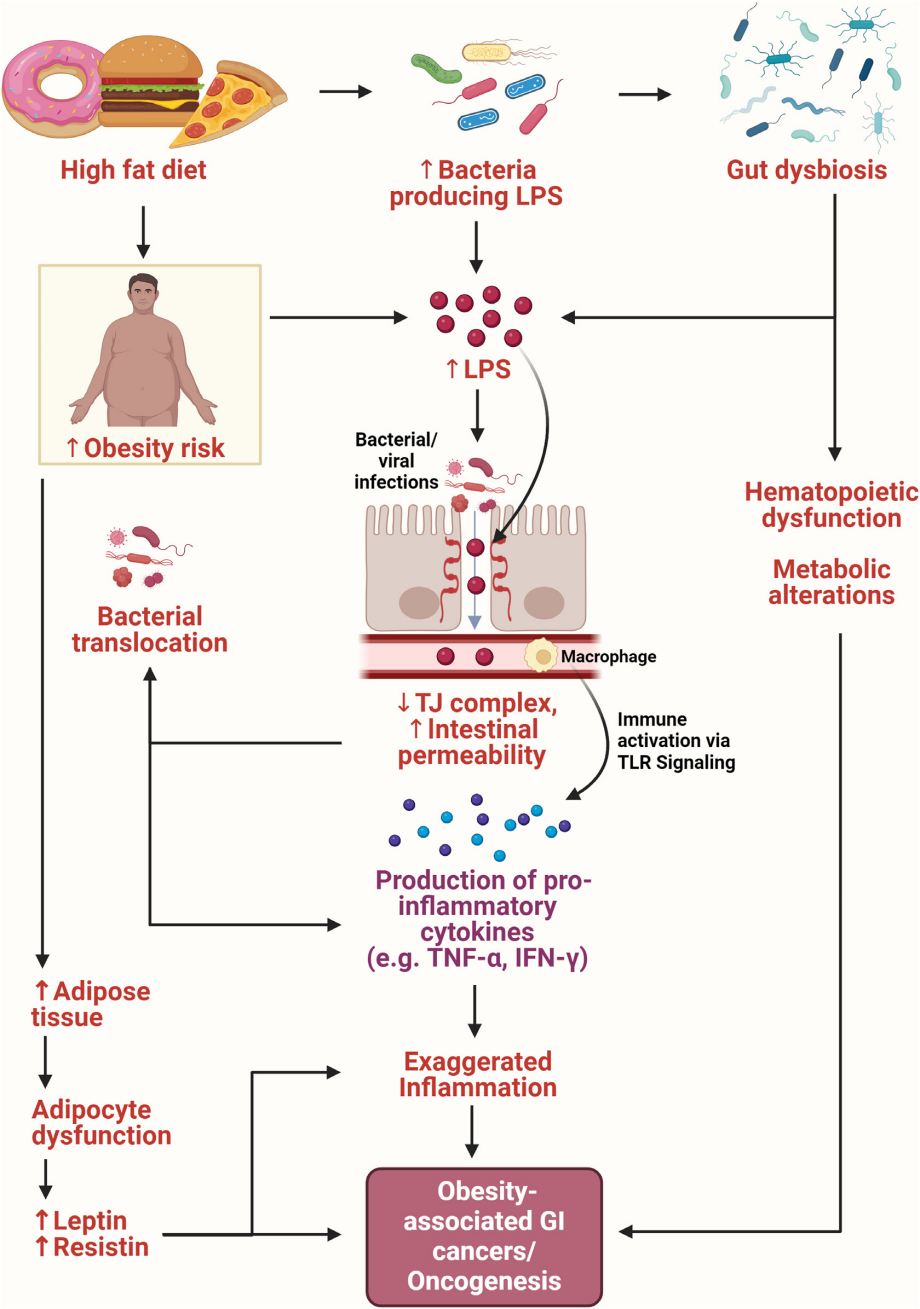
Interconnections of gut microbiome and obesity-associated cancer. The interplay between diet, adipose tissue, gut microbiota dysbiosis, immune system and intestinal permeability leads to the development of obesity-induced gastrointestinal cancer. High-fat diet induces alterations in gut microbiota composition (gut dysbiosis) that increases the population of LPS-producing bacteria in the gut. This results in the downregulation of the expression of TJ proteins, leading to increased permeability of intestinal epithelial cells. Consequently, the defective intestinal barrier increasingly translocate LPS as well as bacteria and viruses. In circulation, LPS binds to macrophages, triggering immune activation via TLR signaling pathways and an upsurge in the release of proinflammatory cytokines such as IL-6, thus inducing inflammation. Additionally, gut dysbiosis induces hematopoietic dysfunction and metabolic alterations. Consumption of a high-fat diet promotes an increase in adipose tissue and fat mass as well as the risk of obesity, disrupting adipocyte function. This leads to increased release of leptin and resistin, which contribute to the amplification of inflammatory signaling pathways. All of these pathways contribute to the development of obesity-associated gastrointestinal cancers by creating an inflammatory environment conducive for tumorigenesis. Thus, this signifies the complex and interconnected relationship between diet, gut dysbiosis, obesity and GI cancers. IL-6, interleukin-6; LPS, liposaccharide; TJ, tight junctions; TLR, Toll-like receptor; TNF-α, tumor necrosis factor alpha.

Perturbations in metabolic pathways can have significant ramifications on the gut environment. Abnormalities in glucose metabolism, the onset of insulin resistance, and a shifted metabolome can contribute to a gut environment that fosters obesity-associated CRC.^191^ The impact of diet, particularly a high-fat diet (HFD), on the gut and CRC cannot be understated. Consuming HFDs increases fat mass and body weight, inflammatory mediators, and blood glucose levels. This heightened inflammatory state is evidenced by an increased prevalence of large polyps in the colon.^192^ Fatty acids, integral to HFDs, have been shown to amplify inflammatory signaling, influencing the inflammatory cascade that potentially leads to tumorigenesis (Fig. 5). Further, those on an HFD regimen had a marked increase in colonic adenomas compared to those on a standard diet, a distinction that was accompanied by elevated levels of serum leptin, insulin, IGF-1, and TNF-α.^193^ HFDs promote CRC through multiple avenues: impairing the gut barrier function, activating microbial imbalances (dysbiosis), and modulating the levels of lysophosphatidic acid, a lipid signaling molecule (Fig. 5).^194^ Yet, hope emerges from interventions and lifestyle choices. Sulindac, a non-steroidal anti-inflammatory drug, has shown promise in adjusting tumor gene expression alongside weight loss. This modulation promotes an immune environment less conducive to tumor growth. Certain fecal microbes, including *Prevotella, Coprobacillus*, and *Akkermansia muciniphila*, have positively correlated with tumorigenesis. Still, their levels were effectively reduced by sulindac in obesity models.^195^ Additionally, regular physical activity emerges as a crucial ally, offering multiple benefits: rectifying the microbial imbalance (dysbiosis), enhancing body composition, and tempering the inflammatory state, thus potentially reducing CRC risk.^196^ Understanding the gut's microbial dynamics can provide invaluable insights into colorectal cancer's onset, progression, and potential preventive measures.

There is a growing appreciation of the intricate relationships between gut microbiota, obesity, and breast cancer development and progression. Several bacterial species have shown differential abundances in breast cancer patients of varying grades. Notably, women with higher-grade breast cancer had increased levels of *Firmicutes sp*., *Actinobacteria sp*., *Proteobacteria sp*., and *Enterobacteriaceae sp*. compared to those with lower-grade breast cancer. Furthermore, a correlation has been observed between BMI and microbial abundance; women with a BMI of ≥ 25 kg/m^2^ presented a significantly higher abundance of *Firmicutes sp*. than their counterparts with a lower BMI.^197^ The implications of maternal health on the infant gut microbiome are profound. Maternal obesity can induce lasting disruptions in the infant's gut microbiome, which may predispose them to health challenges later in life. For instance, breast cancer patients have shown a decreased abundance of SCFA producing gut microbes.^198^ Since SCFAs play a pivotal role in maintaining gut health and systemic immune responses, these alterations could have broader health implications. Maternal obesity can also amplify breast cancer risks for the offspring, mediated by alterations in the gut microbiota. Encouragingly, dietary interventions geared towards enriching SCFA-producing bacteria have shown potential to combat resistance to breast cancer therapies in offspring.^199^ The dietary component, genistein (GE), a derivative of soybeans, has garnered attention for its multi-faceted benefits. GE counteracts obesity-associated breast cancer, modulates the gut microbiome, and introduces favorable epigenetic changes. GE has shown promise in positively reshaping the offspring's gut microbiome when integrated into the maternal diet. This shift is characterized by a marked increase in SCFA-producing bacteria, such as *Bifidobacterium, Bacteroides*, and *Allobaculum.*^200^ In summary, understanding the complex web of interactions between the gut microbiota, dietary interventions, and breast cancer can pave the way for innovative preventive and therapeutic strategies. The multi-layered relationship between obesity, cancer, and the gut microbiome propels us to think if we can harness this knowledge to forge a path toward better health outcomes.

## 5 The impact of obesity on cancer treatment resistance

Obesity is a well-established risk factor for the development and progression of various cancers, including breast, colorectal, pancreatic, and endometrial cancers. However, beyond its role in cancer initiation and progression, obesity also has a profound impact on the effectiveness of cancer treatments. The relationship between obesity and cancer treatment resistance is complex and multifaceted, involving a range of biological, pharmacokinetic, and systemic factors that collectively diminish the therapeutic efficacy and compromise patient outcomes.^201^

### 5.1 Obesity and chemotherapy resistance

One of the most significant challenges in treating obese cancer patients is the reduced effectiveness of chemotherapy. Despite weight-based dosing strategies designed to optimize therapeutic levels, studies have shown that obese patients often exhibit lower response rates to chemotherapy. This is particularly evident in breast cancer, where obese patients are less likely to achieve complete pathological responses and have higher rates of recurrence.^201^ Several factors contribute to this phenomenon:

Pharmacokinetic variations: Obesity alters the pharmacokinetics of chemotherapy drugs, affecting their absorption, distribution, metabolism, and excretion. Increased adipose tissue can lead to altered drug distribution, resulting in suboptimal drug concentrations in the tumor microenvironment. Additionally, obesity-associated changes in liver function can affect drug metabolism, potentially reducing the efficacy of chemotherapeutic agents.^27^^,^^202^

Inflammatory microenvironment: Obesity is associated with chronic low-grade inflammation, characterized by elevated levels of pro-inflammatory cytokines such as IL-6, TNF-α, and C-reactive protein. This inflammatory milieu can promote tumor progression and contribute to chemotherapy resistance by activating survival pathways in cancer cells and enhancing their ability to withstand cytotoxic stress.^16^

Adipocyte-cancer cell interactions: Adipocytes, or fat cells, in the tumor microenvironment can interact directly with cancer cells, secreting factors that promote cancer cell survival and resistance to chemotherapy. These interactions can enhance cancer cell resistance to apoptosis, the programmed cell death that many chemotherapy agents aim to induce.^172^

### 5.2 Impact on targeted therapies and immunotherapy

The influence of obesity extends beyond chemotherapy, also affecting the efficacy of targeted therapies and immunotherapy. One notable example is the diminished response to anti-vascular endothelial growth factor (VEGF) therapies in obese patients. VEGF is a key regulator of angiogenesis, the formation of new blood vessels, which tumors exploit to secure a blood supply and support their growth. Anti-VEGF therapies aim to disrupt this process, but in obese patients, the presence of hypoxic adipose tissue within tumors and the systemic increase in inflammatory cytokines such as IL-6 and FGF2 compromise the effectiveness of these treatments.^222^ Studies in murine models have demonstrated that obesity-associated increases in IL-6 production from both adipocytes and myeloid cells within tumors lead to enhanced tumor hypoxia and angiogenesis, counteracting the intended effects of anti-VEGF therapy.^203^

Similarly, obesity can impact the efficacy of immunotherapies, which harness the immune system to fight cancer. Obesity-induced chronic inflammation and immune dysregulation can lead to an immunosuppressive tumor microenvironment, characterized by increased levels of myeloid-derived suppressor cells (MDSCs) and regulatory T cells (Tregs). These cells inhibit the anti-tumor immune response, reducing the effectiveness of immunotherapeutic agents such as checkpoint inhibitors. Furthermore, the altered metabolism in obese individuals can affect the function and persistence of cytotoxic T cells, which are critical for the success of immunotherapy.^204^

### 5.3 Surgical and radiation therapy challenges

Obesity also presents unique challenges in the context of surgical and radiation therapies. Obese patients are at an increased risk of complications during surgery, including wound infections, delayed healing, and anesthesia-related issues. The increased adiposity can make surgical procedures more technically challenging, leading to longer operation times and potentially incomplete resections, which may leave residual tumor tissue and increase the likelihood of recurrence.^205^

In radiation therapy, the presence of excess adipose tissue can affect the precision and effectiveness of treatment. Obesity can lead to altered body geometry, which may complicate the accurate delivery of radiation doses to the tumor while sparing surrounding healthy tissue. Additionally, the pro-inflammatory state associated with obesity can exacerbate radiation-induced toxicity, further complicating treatment.^206^

### 5.4 Mechanistic insights and future directions

The mechanisms by which obesity contributes to cancer treatment resistance are complex and involve multiple pathways. Key factors include:

Insulin resistance and hyperinsulinemia: Obesity is often associated with insulin resistance, leading to hyperinsulinemia, which can promote tumor growth and survival. Insulin and IGF-1 signaling pathways are frequently upregulated in obese individuals, enhancing cancer cell proliferation and reducing the effectiveness of therapies that target these pathways.^207^^,^^208^

Adipokines and metabolic dysregulation: Adipokines, such as leptin and adiponectin, are hormones produced by adipose tissue that play roles in metabolism and inflammation. In obesity, there is an imbalance in adipokine levels, with increased leptin and decreased adiponectin, contributing to a pro-tumorigenic environment. These changes can influence cancer cell behavior and resistance to therapy.^209^

Epigenetic modifications: Obesity can lead to epigenetic changes that alter gene expression in both cancer cells and the surrounding microenvironment. These modifications can promote resistance to therapy by activating survival pathways or silencing genes involved in drug sensitivity.^210^

In summary, obesity significantly influences cancer treatment resistance, presenting challenges across various therapeutic modalities including chemotherapy, targeted therapies, immunotherapy, surgery, and radiation. The interplay between obesity-induced metabolic changes, chronic inflammation, and the tumor microenvironment creates a context in which cancer cells can thrive despite aggressive treatment. Addressing these challenges requires a multi-faceted approach that includes optimizing treatment strategies for obese patients, developing therapies that specifically target obesity-related mechanisms of resistance, and further research to elucidate the underlying biological processes. By advancing our understanding of how obesity impacts cancer treatment resistance, we can improve outcomes for this growing population of cancer patients.

## 6 Obesity and immunotherapy response

Recent evidence has highlighted a complex and somewhat paradoxical relationship between obesity and immunotherapy outcomes in cancer treatment, particularly in malignancies such as melanoma and non-small cell lung cancer (NSCLC).^211^ This phenomenon, often termed the 'obesity paradox,' refers to the observation that despite obesity being a known risk factor for various cancers and generally associated with poorer prognosis, obese patients may actually exhibit improved responses to immune checkpoint inhibitors (ICIs) such as anti-PD-1/PD-L1 and anti-CTLA-4 therapies.^212^, ^213^, ^214^ Several potential mechanisms have been proposed to explain this paradox.^215^ One hypothesis suggests that the chronic low-grade inflammation characteristic of obesity alters the tumor microenvironment in a way that enhances the recruitment and activation of immune cells. For instance, obesity-related factors such as elevated levels of pro-inflammatory cytokines (e.g., IL-6, TNF-α) and adipokines (e.g., leptin) may promote an immune-stimulatory environment that facilitates a stronger anti-tumor response when ICIs are administered.^216^

Moreover, insulin resistance and hyperinsulinemia, common in obese individuals, could further modulate immune responses, potentially enhancing the effectiveness of ICIs. This could be due to the fact that insulin signaling pathways intersect with immune pathways, thereby influencing the activity of immune cells such as T lymphocytes.^216^ However, it is important to approach these findings with caution. Not all studies agree on the beneficial impact of obesity on immunotherapy. Some research indicates that obesity does not universally confer improved outcomes and that in certain contexts, it may even exacerbate adverse effects or lead to poorer overall survival. This inconsistency in the data underscores the need for further research to clarify the conditions under which obesity might influence immunotherapy efficacy.

Given these mixed findings, clinicians and researchers must consider the individual metabolic and inflammatory profiles of cancer patients when designing and administering immunotherapy regimens. Personalized approaches that take into account not just BMI but also factors like adiposity distribution, metabolic health, and systemic inflammation may be key to optimizing treatment outcomes. Future large-scale studies should aim to disentangle the complex interactions between obesity, the immune system, and cancer therapy to better understand how to leverage the potential benefits of the 'obesity paradox' while mitigating any associated risks.

## 7 Therapeutic interventions targeting the obesity-cancer connection

Obesity and cancer are linked through a complex network of molecular and biochemical pathways, making them promising targets for therapeutic interventions. The development of therapies that address both conditions requires a better understanding of these pathways and their roles in disease progression. Below, we explore key therapeutic strategies that aim to disrupt the obesity-cancer link, focusing on both established and emerging treatments.

### 7.1 Metformin (Glucophage)

metformin (Glucophage), a widely used antidiabetic drug, has gained attention for its potential anti-cancer properties. Clinical and epidemiological studies have consistently shown that metformin (Glucophage) not only helps manage diabetes but also reduces the incidence and mortality of several cancers, including colorectal, pancreatic, hepatocellular, and breast cancers. The anti-cancer effects of metformin (Glucophage) are thought to result from a combination of direct and indirect mechanisms, primarily mediated through the activation of AMPK. metformin (Glucophage) (Glucophage) enters cells via organic cation transporter 1 (OCT1) and accumulates in mitochondria, where it inhibits complex I of the mitochondrial respiratory chain. This inhibition increases intracellular AMP levels, leading to the activation of AMPK through phosphorylation by liver kinase B1 (LKB1) on threonine 172.^217^^,^^218^ AMPK activation plays a crucial role in metabolic regulation, including the suppression of hepatic gluconeogenesis, which helps normalize fasting blood glucose levels, reduce circulating insulin, and improve insulin sensitivity.^219^ Lower insulin levels can diminish the proliferative and anti-apoptotic signals in cancer cells that exhibit insulin resistance, thereby potentially inhibiting cancer progression.^220^

Moreover, metformin (Glucophage) has demonstrated significant effects in non-diabetic breast cancer patients. A six-month regimen of metformin (Glucophage) led to a 22 % reduction in insulin levels, along with decreases in glucose, leptin, and body weight. These metabolic changes were accompanied by a reduction in C-reactive protein, a marker of chronic inflammation, and downregulation of key oncogenic pathways, such as AKT and MAPK, in tumor tissues.^221^ The inhibition of these pathways is believed to be mediated by AMPK, which stabilizes tuberous sclerosis complex 2 (TSC2) and inhibits the mammalian target of rapamycin complex 1 (mTORC1). This inhibition downregulates p70S6 K and 4EBP1, key regulators of protein synthesis and cell proliferation.^213^ However, it is important to note that the doses of metformin (Glucophage) used *in vitro* often exceed those that are safely tolerable in humans, which limits the direct translatability of these findings to clinical practice.^222^

### 7.2 Adiponectin

Adiponectin, a hormone produced by adipose tissue, has gained recognition for its antiproliferative, proapoptotic, and antiangiogenic properties, making it a potential therapeutic target in the obesity-cancer connection. Adiponectin levels are inversely correlated with the risk of several cancers, including endometrial, breast, colon, and prostate cancers.^126^^,^^142^ The precise mechanisms by which adiponectin exerts its anti-cancer effects are still under investigation, but it is thought to deactivate key signaling pathways involved in tumor growth, such as mitogen-activated protein kinase (MAPK) kinases 1 and 3, as well as extracellular signal-regulated kinases 1 and 2 (ERK1/2).^223^ Additionally, adiponectin enhances insulin sensitivity and reduces inflammation, both of which are critical in mitigating the obesity-cancer link. The hormone's anti-inflammatory properties are particularly important, as chronic inflammation is a well-known contributor to cancer development and progression.^106^ By modulating these pathways, adiponectin presents a dual benefit in both improving metabolic health and reducing cancer risk.

### 7.3 Targeting epigenetic modifications

Emerging evidence has highlighted the role of fat mass and FTO in the regulation of m6A RNA methylation, a process that influences gene expression and is implicated in both obesity and cancer. Inhibiting FTO has shown promise in reducing obesity and combating cancer by modulating the FTO-m6A-YTHDF2 axis.^224^ For example, catechin EGCG, an anti-obesity compound found in green tea, has been identified as an FTO inhibitor that can reduce fat mass and improve glucose metabolism, while also exerting anti-cancer effects.^225^ Entacapone, another FTO inhibitor, has demonstrated potential in reducing body weight and glucose levels, as well as in inhibiting the growth of various cancers. It is currently being explored for its role in post-cancer treatment to prevent secondary tumor growth.^226^ Additionally, FTO inhibitors such as CS1 (Bisantrene) and CS2 (Brequinar) have been shown to decrease the self-renewal capacity of leukemia stem/initiating cells (LSCs/LICs), highlighting their potential use as monotherapies or in combination with hypomethylating agents for treating refractory and relapsed cancers.^227^^,^^228^ These findings suggest that FTO-targeting strategies could serve as potent therapeutic tools in addressing both obesity and cancer.^229^

### 7.4 Anti-inflammatory interventions

Chronic inflammation is a key driver of both obesity and cancer, making anti-inflammatory interventions a crucial aspect of therapeutic strategies. The reduction of systemic inflammation can be achieved through various means, including lifestyle interventions (e.g., diet and exercise), pharmacological agents (e.g., nonsteroidal anti-inflammatory drugs), and natural compounds (e.g., omega-3 fatty acids, curcumin). These interventions not only improve metabolic health but also reduce the pro-inflammatory environment that supports tumor growth and progression. In summary, the therapeutic strategies targeting the obesity-cancer connection are diverse and continue to evolve as our understanding of the underlying mechanisms deepens. metformin (Glucophage), adiponectin, FTO inhibitors, and anti-inflammatory interventions represent promising approaches to disrupt the biochemical pathways that link obesity and cancer. As research progresses, optimizing these therapies for clinical use will be crucial in improving outcomes for patients with obesity-related cancers. Ultimately, the successful integration of these strategies into clinical practice could offer significant benefits in both oncology and metabolic health, paving the way for more effective and personalized treatments.

## 8 Conclusion and future perspectives

In conclusion, the escalating prevalence of obesity presents a significant public health concern closely linked to various types of cancer. This association stems from obesity-induced adipose inflammation, which triggers the secretion of proinflammatory factors, systemic metabolism dysregulation, and alterations in the TME, thereby promoting tumorigenesis. White adipose tissue, in particular, serves as a critical mediator in obesity-associated carcinogenesis and tumor progression. Changes in the TME, such as insulin resistance, elevated proinflammatory mediators, and dysregulated adipokine levels, are tied to white adipose tissue inflammation. Given the strong correlation between obesity-related changes and increased cancer risk, targeting adipose tissue inflammation holds promise for addressing both obesity and cancer. Strategies that attenuate white adipose tissue inflammation, such as utilizing metformin (Glucophage) and targeting the MAPK pathway, may prove more effective than those that do not account for the obesity-associated cancer burden. Such therapeutic approaches could potentially be combined with healthy low-calorie diets for obese individuals with tumors and used as adjuvant treatments during cancer chemotherapy in clinical trials.

Moreover, recognizing white adipose tissue inflammatory status irrespective of BMI may provide a more comprehensive understanding of the pathophysiological consequences of obesity. Identifying an adipose inflammation biomarker that can be feasibly implemented in clinics and examining the link between obesity and various cancers more broadly could offer crucial insights. Further investigations are needed to elucidate the primary inflammatory mechanisms and pinpoint effective targets and therapies while facilitating early diagnosis and improved prevention for both obesity and cancer. This can be achieved through a more in-depth examination of the cellular, molecular, and metabolic mechanisms linking obesity, inflammation, and cancer.

Finally, maintaining a balance between adipocytes and immune cells is crucial for normal body metabolism. Exploring immune cells and pathways in greater detail may help uncover the connections between obesity, inflammation, and cancer, ultimately paving the way for novel therapeutic interventions and improved patient outcomes. By advancing our understanding of these complex interactions and developing targeted therapies, we may be able to effectively combat the growing global health burden of obesity and cancer. The limitations of narrative reviews could be introducing selection bias due to the subjective process of study selection and the potential for unequal consideration of evidence. The conclusions drawn in narrative reviews should be interpreted cautiously, particularly when comparing them to the more robust conclusions derived from systematic reviews. Future research in this area would benefit from a systematic review that applies rigorous methodologies to evaluate the quality and strength of the evidence linking obesity and cancer. Such an approach would complement our narrative review by providing a more structured and critical appraisal of the available literature.

## Declaration of competing interest

The authors declare that they have no known competing financial interests or personal relationships that could have appeared to influence the work reported in this paper.
